# The relevance of oral exposure in the workplace: a systematic review and meta-analysis

**DOI:** 10.3389/fpubh.2023.1298744

**Published:** 2023-11-30

**Authors:** Marlene Dietz, Wiebke Ella Schnieder, Urs Schlüter, Anke Kahl

**Affiliations:** ^1^Unit 4.I.4 Exposure Assessment, Exposure Science, Division 4 Hazardous Substances and Biological Agents, Federal Institute for Occupational Safety and Health (BAuA), Dortmund, Germany; ^2^America Chair of Occupational Safety, School of Mechanical Engineering and Safety Engineering, University of Wuppertal, Wuppertal, Germany; ^3^Environmental Monitoring and Forensic Chemistry, Hamm-Lippstadt University of Applied Sciences, Hamm, Germany

**Keywords:** inadvertent ingestion, oral exposure, occupation, workplace, overall exposure, PRISMA

## Abstract

**Introduction:**

The inclusion of all relevant exposure routes in the exposure assessment is essential for the protection of workers. However, under European chemical regulations but also for workplace risk assessments according to occupational safety and health (OSH) requirements, the quantitative assessment of oral exposure is usually neglected assuming good occupational hygiene. In contrast, several studies point to the importance of unintentional ingestion in the workplace. To our knowledge, there is no systematic analysis of the extent of this exposure route.

**Methods:**

Therefore, the aim of this study was to assess systematically the current knowledge on the relevance of occupational oral exposure using the Preferred Reporting Items for Systematic Reviews and Meta-Analysis (PRISMA) method. Five electronic databases and nine institutional websites were searched for all publications on the relevance. The data were extracted into a concept matrix. In the subsequent meta-analysis, the identified conclusions on the relevance were analyzed. In addition, the measurement methods or modeling approaches that were described for occupational oral exposure were determined as well as the potentially relevant workplaces and substances.

**Results:**

In total, 147 studies were included in this analysis that contain a general or several, differentiated assessments of the relevance of occupational oral exposure. Nine of these studies assessed this exposure route as irrelevant. However, 123 studies considered oral exposure as potentially contributing and 80 studies explicitly identified it as relevant. 78 and 94 of the publications described modeling and measurement approaches, respectively. The workplaces frequently identified as potentially or explicitly relevant were other indoor, other industrial or recycling workplaces. Analogously, metals, dust and powders or pesticides were the most frequently investigated substance groups.

**Discussion:**

As several studies assessed occupational oral exposure as relevant in the context of different workplaces and substances, further investigation of this exposure route is needed. This systematic review and meta-analysis serve as a basis for further development of feasible assessment methods for this route of exposure.

## 1 Introduction

The assessment of all contributing exposure pathways is fundamental for the protection of workers. To date, European chemical regulations (1, 2) or workplace risk assessments under OSH (3) have focused on the assessment of inhalation and dermal exposure. However, oral exposure in the workplace may be a third contributing exposure pathway and depends on several aspects. First, it is influenced by the workers behavior and their compliance with hygiene practices (4–6). For example, the occurrence of oral exposure may depend on the frequency of hand washing during a shift (7). Second, exposure sources such as spray deposition or contaminated surfaces, objects or skin serve as starting points for oral exposure (5, 8). Third, other influences such as the transfer efficiency of substances between hand and mouth (6, 9, 10) and fourth, the nature of a substance such as metals versus infectious agents influence the formation of oral exposure (11).

However, there are different perspectives on oral exposure in the workplace. The REACH Regulation states in its guidance document R.14 that oral exposure only needs to be considered in specific cases. It specifies that compliance with good occupational hygiene practices is usually sufficient to address oral exposure and that no quantitative assessment of unintentional ingestion is needed (12).

In contrast to this, Cherrie et al. estimated that 15.6% of the total UK working population is exposed by inadvertent ingestion (11). In addition, studies comparing external and internal exposure of workers, hint on a potential relevance of occupational oral exposure. In these studies, dermal and inhalation exposure measurements and biomonitoring were performed in parallel. For example, Beattie et al. examined exposure to nickel and hexavalent chromium exposure in the electroplating industry and found that the corresponding biomonitoring levels could not be explained by inhalation and dermal exposure levels, only (13). The authors concluded that ingestion might contribute to the total exposure of workers. In addition, Karita et al. studied the exposure of workers in a lead refinery and demonstrated a positive correlation between external facial or nail exposure and blood lead levels (*r* = 0.730 and *r* = 0.590, respectively) (14). However, the dermal uptake of lead and the inhalation uptake of the present particle sizes were negligible.

Consequently, oral exposure in the workplace could contribute significantly to the total exposure of workers. As total exposure needs to be considered for the overall protection of workers, the aim of this systematic review was to investigate the relevance of oral exposure in the workplace by examining published studies.

## 2 Methods

To determine the current state of knowledge and to comprehensively identify publications on the relevance of oral exposure in the workplace, a systematic literature review was conducted using the following PRISMA-based (15) method. No review protocol was defined and the review was not registered.

### 2.1 Information sources and search strategies

The five databases selected as information sources were Web of Science, PubMed, COCHRANE, bergischbib, and Deutsche Nationalbibliothek. Since many project reports are only available on the corresponding institutional websites, nine websites of institutes performing potentially relevant research were added as summarized in Table 1.

**Table 1:** Overview of included databases and institutional websites with corresponding URL.

Information source	Database	Website	URL
Bergischbib	x		http://www.bergischbib.de/
COCHRANE	x		https://www.cochranelibrary.com/advanced-search
Deutsche Nationalbibliothek	x		https://katalog.dnb.de
PubMed	x		https://pubmed.ncbi.nlm.nih.gov/advanced/
Web of Science	x		https://www.webofscience.com
Federal Institute for Occupational Safety and Health (BAuA)		x	https://www.baua.de/DE/Angebote/Publikationen/Publikationen_node.html
United States Environmental Protection Agency (EPA)		x	https://www.epa.gov/nscep (advanced search)
Health and Safety Executive (HSE)		x	https://www.hse.gov.uk/pubns/
Institute of Occupational Medicine (IOM)		x	https://www.iom-world.org/research/online-library/
National Institute for Occupational Safety and Health (NIOSH)		x	https://www2a.cdc.gov/nioshtic-2/advsearch2.asp
Organisation for Economic Co-operation and Development (OECD)		x	https://www.oecd-ilibrary.org/
National Institute for Public Health and the Environment (RIVM)		x	https://www.rivm.nl/en/recentpublications
Netherlands Organisation for Applied Scientific Research (TNO)		x	https://repository.tno.nl/islandora/search/
World Health Organization (WHO)		x	https://apps.who.int/iris/

Since the focus of this literature search was to identify publications on the relevance of occupational oral exposure, all searches included the topics “occupation”, “oral”, and “exposure” as a base search. This base search strategy was specified on the basis of four different subject areas, which all can be used to draw conclusions about oral exposure. This approach resulted in four search strategies, all of which included the base search strategy and addressed the relevance of oral exposure in the workplace from different perspectives:

1. Direct statements on the relevance of oral exposure in the workplace
2. Conclusions based on estimates or modeling approaches
3. Conclusions based on measurements
4. Statements based on activities or substances for which the occurrence of oral exposure was considered relevant in advance.

In the authors’ experience, the database on the relevance of occupational oral exposure to liquids is limited. Therefore, to ensure comprehensive inclusion of liquid-specific literature on the relevance of oral exposure and to verify this lack of research, liquid-specific information was specifically included in the search by combining the first three general search fields with a liquid-specific term. The fourth search field was not further specified as it already covers substance or activity specific publications.

For each of the liquid-specific and general search fields, search terms and corresponding synonyms were identified independently by two of the authors (MD, WS). The results were discussed and combined into applicable search strings by consensus.

For the evaluation of the developed search strings, 15 already known publications were selected that refer to the relevance of oral exposure in the workplace. To test whether the search strings were able to identify these known publications, the search strings were then applied to the Web of Science and PubMed databases, as these contain eight and eleven of the selected evaluation publications, respectively. By analyzing the obtained search results, the search strings were iteratively improved and specified to maximize the number of evaluation publications covered and to minimize the number of irrelevant publications. The developed search strings identified eight of eight available known publications in Web of Science. In PubMed ten of eleven available publications were identified by the search strings. More information on the evaluation is documented in Supplementary Tables S1, S2. The final seven search strings are included in Table 2.

**Table 2:** Applied seven search strings.

No.	Field	Liquid	String
-	*Base term*	n.a.	(Occupa* OR Job OR Employ* OR Profession* OR Worker OR Workplace OR Industr*) AND (Oral* OR Ingest* OR inadvertent OR incidental OR perioral* OR peri-oral*) AND (Expos* OR Intake OR Uptake OR Ingest*)
1	1	No	*Base term* AND (Relevan* OR Importan* OR Significan* OR Critical* OR Essential*)
2	2	No	*Base term* AND (Estimat* OR Calculat* OR Assess* OR Evaluat* OR Rate OR Rating OR Model* OR Explor* OR Measur* OR Monitor*)
3	3	No	*Base term* AND (Biomonitor* OR Total body burden OR Bio-monitor* OR (dermal AND inhalative AND (biomonitor* OR bio-monitor*)))
4	1	Yes	*Base term* AND (Relevan* OR Importan* OR Significan* OR Critical* OR Essential*) AND (liquid OR fluid OR liquor OR Solution OR spray)
5	2	Yes	*Base term* AND (Estimat* OR Calculat* OR Assess* OR Evaluat* OR Rate OR Rating OR Model* OR Explor* OR Measur* OR Monitor*) AND (liquid OR fluid OR liquor OR Solution OR spray)
6	3	Yes	*Base term* AND (Biomonitor* OR Total body burden OR Bio-monitor* OR (dermal AND inhalative AND (biomonitor* OR bio-monitor*))) AND (liquid OR fluid OR liquor OR Solution OR spray)
7	4	n.a.	(Oral* OR Ingest* OR inadvertent OR incidental OR perioral* OR peri-oral*) AND (Expos* OR Intake OR Uptake) AND (spray* OR paint* OR pesticide OR weld* OR print* OR lead OR electroplat*) AND (Occupa* OR Job OR Employ* OR Profession* OR Worker OR Workplace)

In the search strategies, the term “*Base term*” needs to be replaced by its definition from the first row.

The applicability of these search strings to websites was limited because some websites do not necessarily allow complex search strings. Therefore, simplified search strings were used on websites, as documented in Supplementary Table S3. The date of the last search is also documented in this table.

### 2.2 Study selection

For the study selection, criteria were defined for the study population (P) and the study outcome (O). Since a two-step selection process was used, the criteria were defined for a title and abstract and a full text screening level. For both screening levels, the study population P had to be workers. This excluded the most common information on oral exposure of children. At the title and abstract level, the outcome O was sufficiently covered by the description of a study design that generally allows conclusions on the relevance of occupational oral exposure. This was narrowed down at the full text screening level, where the outcome O had to be a specific conclusion on the relevance of occupational oral exposure. In accordance with the four search fields, it was not specified whether this is stated directly or inferred from estimates, measurements or substance- or activity-specific information.

Only studies with a publication date between 2000 and 2023 were included in order to reasonably limit the number of studies identified with respect to the extensive search strings. Except for the databases Deutsche Nationalbibliothek and bergischbib, where mainly German language publications are included and where additional analogous German search strings were used, publications had to be in English.

Search results were evaluated against the defined population and outcome criteria. Each assessment was performed by one author, with two of the authors (MD, WS) working in parallel. To ensure reliable assessments within and between these two authors, a consistency check was performed both for the application of the title and abstract criteria and the full text criteria. Both authors applied the criteria to the same sample of 50 (title and abstract) or 10 (full text) randomly selected publications. Then, the authors’ decisions on the population and outcome criteria were compared, discussed and concluded by consensus. If necessary, a further result of this discussion could be an adoption of the population and outcome criteria. The comparison of the ratings was formalized by the calculation of Cohen’s kappa, which assesses the consistency of the ratings taking into account the consistency that would be expected by chance (16).

During the screening process the publications were sorted according to the occurrence of the keywords “occupation”, “worker”, “workplace”, “occupational exposure”, “ingestion”, and “oral exposure”.

### 2.3 Data extraction and data analysis

A concept matrix was used to extract relevant data from the included publications. This table contains detailed concepts, each representing possible information from the publications (17). For example, “Oral exposure in the workplace is irrelevant because of good hygiene practices” would be a detailed categorization of information from one or more publications. Using this concept, a comprehensive matrix was created by filling in all included publications. The advantage of this method is the very detailed and structured processing of qualitative or quantitative information.

Preparing the final concept matrix, the qualitative information on the relevance of oral exposure in the workplace was extracted into the course categories “irrelevant,” “potentially relevant” and “relevant.” Separately, the categories “conclusion based on modeling” or “conclusion based on measurements” were assessed to evaluate the source of information. This process was performed in parallel by two of the authors (MD, WS).

Subsequently, one of the authors (MD) developed concepts that are more detailed. A distinction was made between whether a conclusion was drawn in the publication reviewed or whether the publication reviewed included this conclusion as a citation. In the second case, it was checked whether the primary publication is included in this PRISMA study and, if so, the citing study was not counted as a new statement on the relevance of occupational oral exposure. If the primary publication was not included in this PRISMA study, the citing study identified during this review remained in the evaluation. This procedure avoids overweighting the conclusions of single studies. Due to this approach and the extensive search strategies and databases/websites, no further publications were identified based on the references of included studies.

Based on this categorization, the frequency of different conclusions on oral exposure in the workplace and the possibly underlying modeling or measuring approaches were analyzed. In particular, the dependencies between the workplace, the substance group and the relevance rating were investigated. Furthermore, modeling and measurement approaches underlying the conclusions were disclosed. The risk of a bias was discussed qualitatively.

### 2.4 Software

During the progress of the searches, the software EndNote X9 (18) was used for literature management. CADIMA 2.2.4.2 (19) was used for the automatic removal of duplicates, which was refined manually to also delete for example the same publications with differently abbreviated journal names. Additionally, the consistency checks, the title and abstract and the full text screening were performed with CADIMA as supportive software. The development of the concept matrix was carried out in Excel 2016 (20) and the further evaluation was performed in R 4.2.3 (21).

## 3 Results

### 3.1 Study selection and included studies

Cohen’s kappa was used to evaluate the agreement of authors in identifying the publications during the consistency checks for title and abstract and full text screening. The obtained values were 0.627 and 0.814 which can be considered as substantial agreement and almost perfect agreement according to Landis and Koch (22). Therefore, neither the title and abstract criteria nor the full text criteria needed to be adjusted.

**Figure 1:**
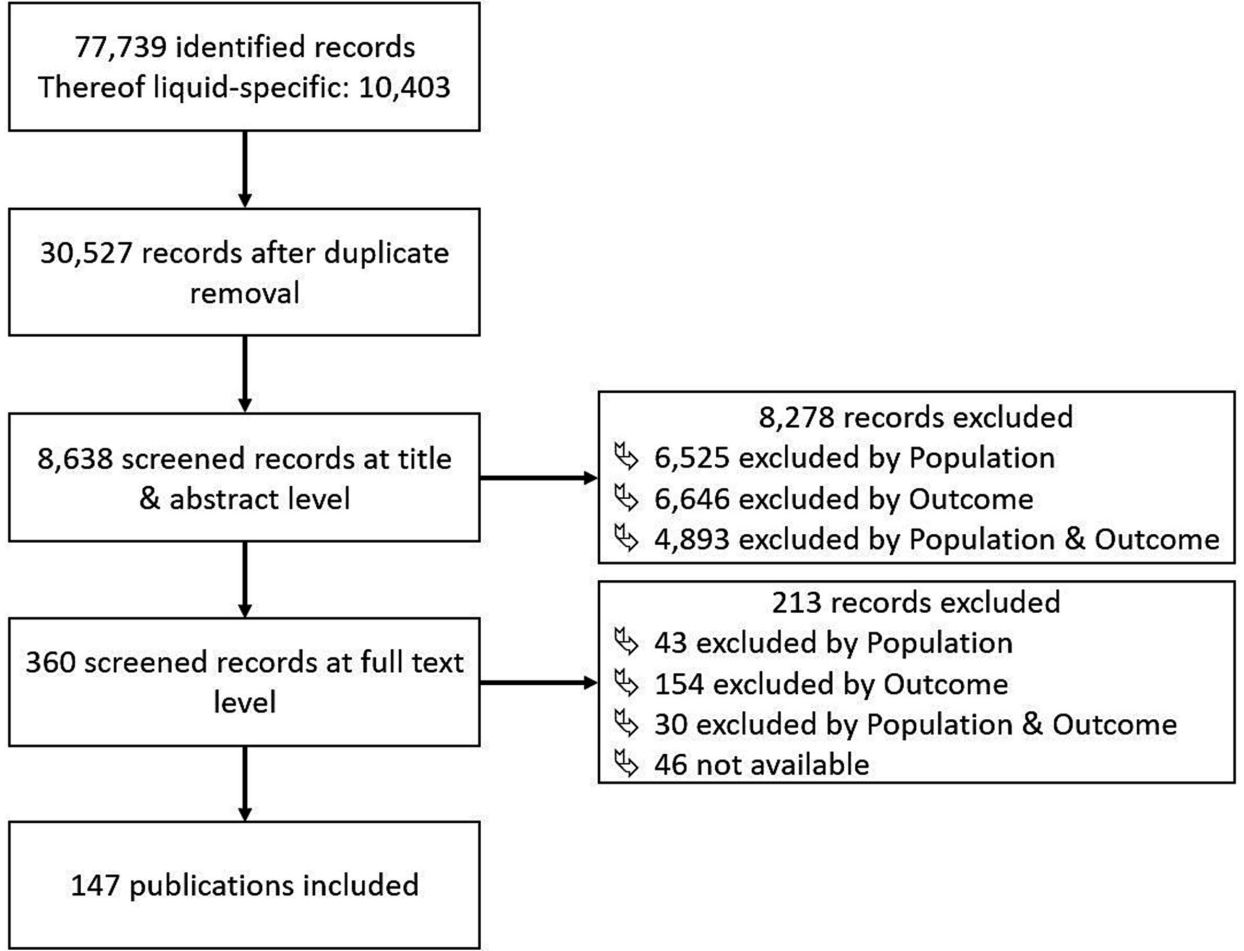
Flowchart including number of identified liquid-specific records and negative ratings for population and outcome criteria.

By applying the described methodology, a total of 77,739 publications were identified, 10,403 of which resulted from the liquid-specific search strategies 4–6. This highlighted the limited database for liquids compared to solids and in the following liquid-specific and non-specific publications were considered together. After removing duplicates, 30,527 records remained. Since this number could not be fully screened by the authors at the title and abstract level, the first 8,638 (28.3%) publications were examined, after the publications were sorted according to their relevance as described in the methods. Throughout the title and abstract screening process, fewer and fewer relevant publications were identified. Therefore, it was assumed that most relevant publications have been screened. Of the 8,638 records, 8,278 were excluded because of negative ratings of the population and outcome criteria in 6,525 and 6,646 cases, respectively. Of these, 4,893 records were negative for both, population and outcome criteria. 360 publications were identified for consideration at the next screening stage. After the title and abstract screening, the full texts of these remaining 360 publications were all screened, resulting in the inclusion of 147 publications. From the excluded records, 43 and 154 publications did not fulfil the population and outcome criteria, respectively. 30 records did neither fulfil population nor outcome criteria. This screening process was summarized in the form of a flowchart in Figure 1, which additionally documents the reasons for exclusion. A list of the included studies with the extracted information for the workplace, the substance group and the categorization of relevance is provided in Supplementary Table S4.

**Figure 2:**
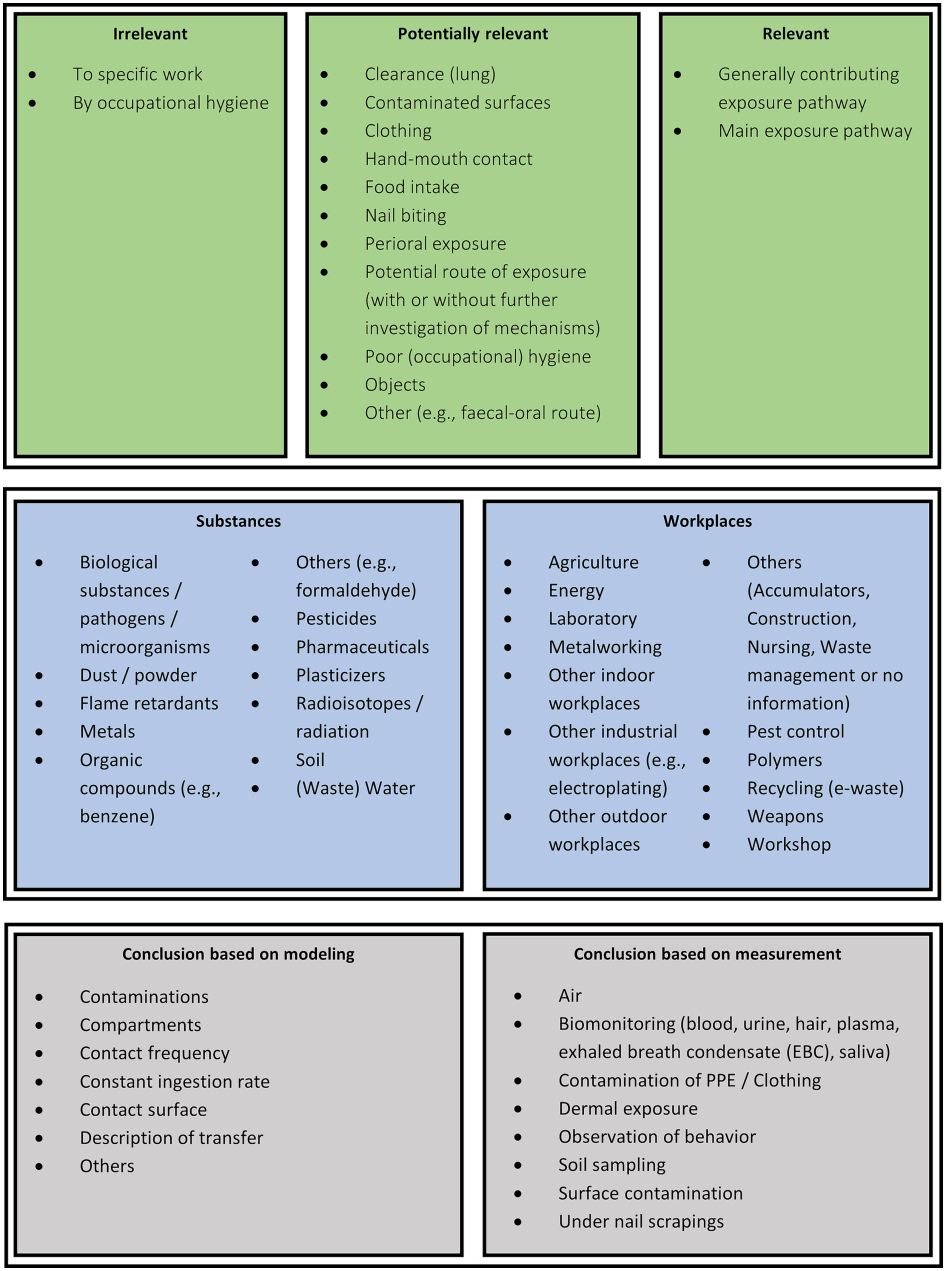
Overview of detailed categories for relevance, substances, workplaces and conclusions based on models or measurements.

Detailed categories were developed to extract and summarize the information from the 147 included publications. In addition, information on the substance and the workplace was extracted in the form of detailed categories as summarized in Figure 2. Where categories overlapped, the most descriptive category was chosen to provide the most accurate picture. For example, agricultural work would be categorized as “Agriculture” rather than “Other outdoor workplaces”. If more than one category was needed to specify, e.g., the substance, those categories were selected. For example, an investigation of metal-contaminated dusts would result in the categories “Metals” and “Dust / powder”.

**Figure 3:**
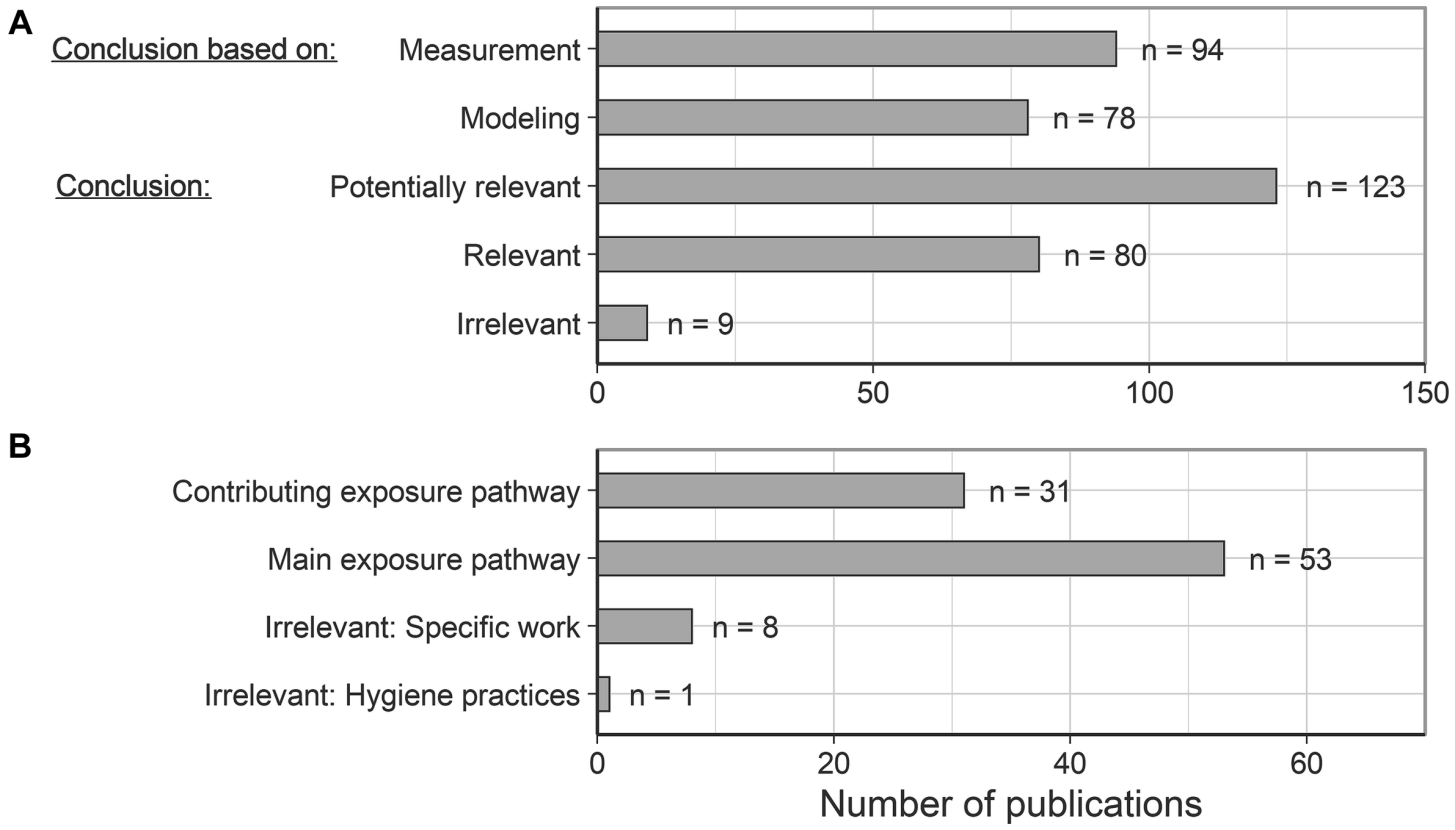
**(A)** Overview of the information bases and conclusions of the included publications on occupational oral exposure. **(B)** Detailed investigation of different conclusions on the relevance of occupational oral exposure.

Thus, 147 publications made statements about the relevance of oral exposure in the workplace. As shown in Figure 3A, 94 and 78 of these statements were based on monitoring or modeling approaches, respectively. In 123 of the 147 publications, the authors concluded that oral exposure could potentially be relevant for the total exposure of workers. This statement was narrowed down in 80 publications to the conclusion that oral exposure was a definite contributor. Only the authors of nine publications concluded that occupational oral exposure was not relevant. Since in this evaluation publications can be classified into several categories (e.g., when statements are concretized for specific workplaces) the sum of all entries in this evaluation was larger than 147.

### 3.2 Conclusions on oral exposure in the workplace

The conclusions on the relevance of occupational oral exposure were examined in some more detail here (see also Figure 3B). Of the 80 publications that considered occupational oral exposure to be relevant, 53 considered it to be the main exposure pathway and 31 considered it to be at least one of the contributing pathways next to dermal and/or inhalation exposure. The authors of nine publications mentioned oral exposure as an occupational exposure pathway but considered the investigated situation as irrelevant for oral exposure: one publication due to compliance with good hygiene practices, describing the use of plasticizers in the production of polymers (23); eight due to the specific work considered in these cases (e.g., applications in agriculture or laboratories).

### 3.3 Identification of relevant workplaces

**Figure 4:**
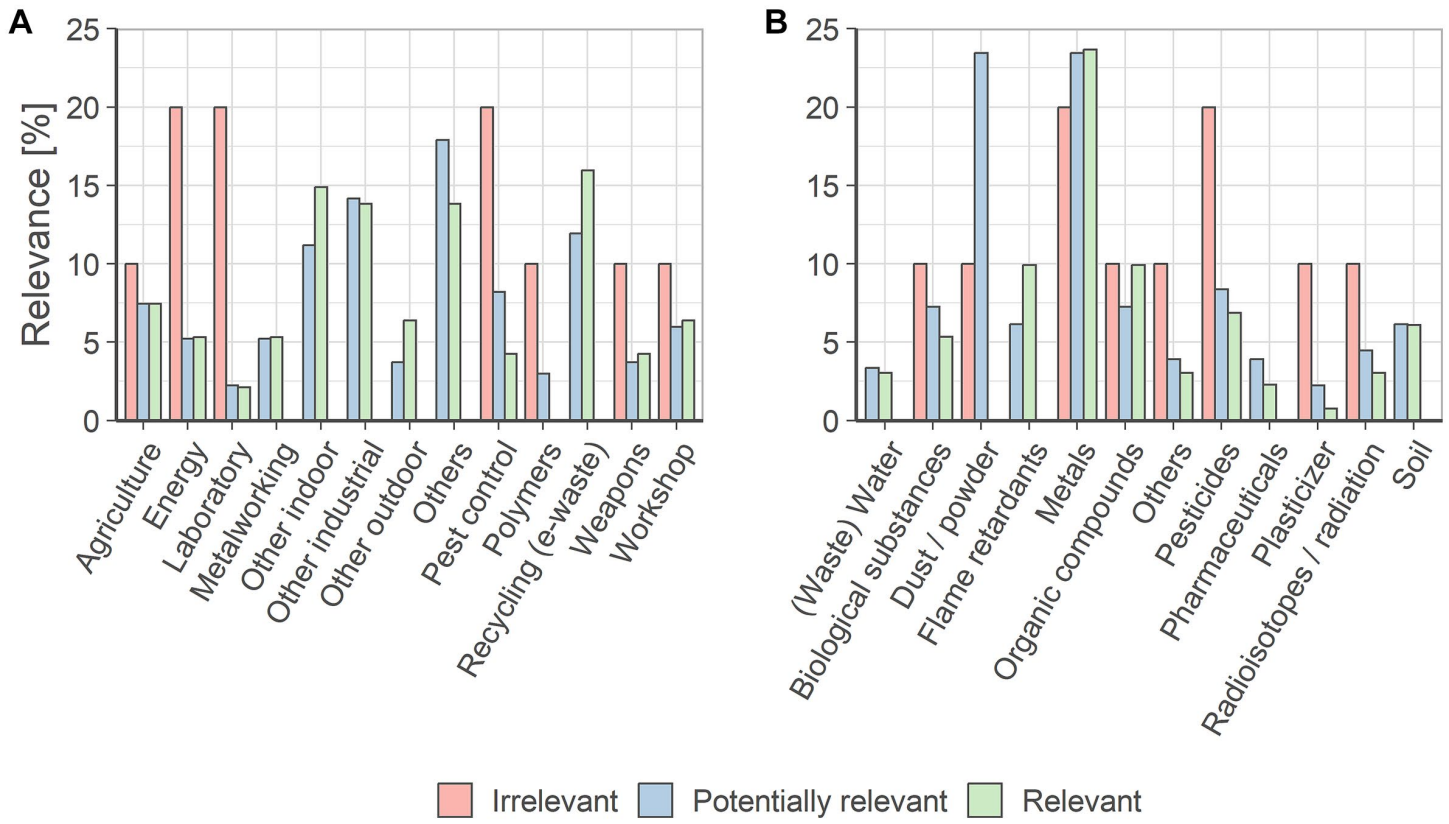
**(A)** Evaluation of the statements on the relevance of occupational oral exposure specific to workplaces. The percentage of relevance relates the respective workplace-specific relevance conclusion to the total number of this conclusion. **(B)** Evaluation of the statements on the relevance of occupational oral exposure specific to substance groups. The percentage of relevance relates the respective substance-specific relevance conclusion to the total number of this conclusion.

In the following, the results on the relevance of oral exposure were related to types of workplaces. The aim was to identify workplaces where oral exposure should be considered because of its relevance. This was done by calculating how often each conclusion applied to a workplace. For example, the nine publications that concluded that occupational oral exposure was not relevant described 10 workplaces, two of these were pest control workplaces. By calculating this proportion for all relevance statements and workplaces, Figure 4A was obtained.

As shown in Figure 4A, the energy sector, laboratory workplaces and pest control workplaces had the highest proportion of statements concluding that occupational oral exposure was irrelevant. However, the significance of the results for irrelevant workplaces was limited because this assessment was based on only 10 different conclusions and a 20% evaluation result corresponded to only two underlying conclusions.

When assessing (potential) relevance, energy, laboratory, metalworking, other outdoor workplaces, polymers, weapons or workshops were less frequently named in the context of (potentially) relevant workplaces. In contrast to this, other indoor workplaces, other industrial workplaces and recycling workplaces were stated (potentially) relevant in over 10% of the corresponding evaluations. Thus, these workplaces were most frequently identified as relevant for occupational oral exposure.

### 3.4 Identification of relevant substances

Since for future assessments not only workplaces but also relevant substances have to be identified, an analogous evaluation of the conclusions on the relevance of occupational exposure and the different substance groups was carried out. Here, for example two of the 10 irrelevant conclusions applied to metals. Figure 4B shows the evaluation results of the different conclusions for substances.

In contrast to the workplace-specific evaluation, the differences between irrelevance and (potential) relevance evaluations were limited in the substance specific-evaluation. This indicated that the current state of knowledge did not allow generalizing conclusions about the relevance of individual substance groups. Instead, Figure 4B shows that the focus of recent research was on dusts and powders, metals and pesticides. Therefore, it was not possible to consider liquids separately for the relevance of occupational oral exposure on this data basis.

### 3.5 Underlying model and measurement approaches

Because some of the search strategies focused on conclusions about the relevance of occupational oral exposure based on modeling or monitoring, the different underlying approaches were summarized to provide an aggregated view of the information sources.

**Figure 5:**
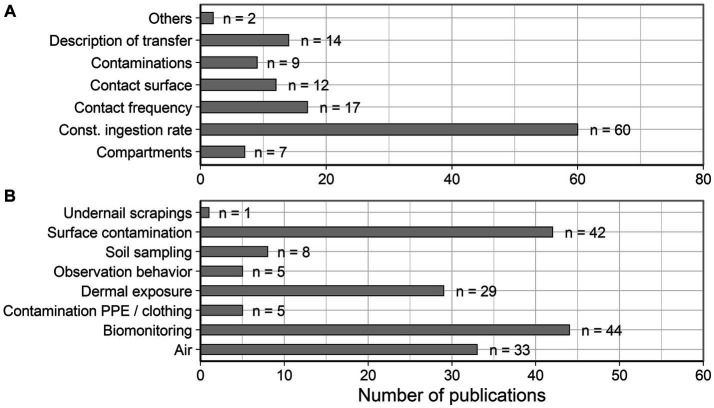
**(A)** Summary of modeling approaches underlying the conclusions on the relevance of occupational oral exposure. **(B)** Summary of measurement approaches underlying the conclusions on the relevance of occupational oral exposure.

Figure 5A illustrates that most calculation-based conclusions used a constant ingestion rate to estimate occupational oral exposure. Other common modeling approaches included the contact frequency, the corresponding contact surface, contaminations and compartments. The description of the transfer of contaminations between compartments was also an underlying concept. Less frequently, the personal behavior and calculations based on biomonitoring results were documented. In particular, the frequency of hand washing was not considered in any of the publications. Hand washing could lower the oral exposure by reducing the previous dermal exposure of the hands. Therefore, personal behavior and biomonitoring evaluations were summarized under the term “Others”.

In Figure 5B, a similar overview is prepared for the underlying measurement approaches.

Corresponding to the data used in the identified modeling approaches, the most common measurement approaches described the investigation of surface contaminations, dermal exposure measurements, biomonitoring and air monitoring. Consequently, the most frequently documented approaches can be used for parallel measurements, as described in the introduction. Less frequently described were undernail scrapings (*n* = 1), saliva analysis (*n* = 2) or exhaled breath condensate analysis (*n* = 3), which were partially summarized under the category “Biomonitoring”. In addition, soil sampling, behavioral observations and analysis of contaminations on PPE or clothing were documented. Furthermore, 30 publications concluded on the relevance of occupational exposure without modeling or measurements, i.e., based on the judgment of the author.

Both, the overview on modeling and measurement approaches showed that complex information and considerable effort are currently required to investigate the oral exposure for specific workplaces. There are no standardized methods for measurement or modeling.

### 3.6 Analysis of bias

Bias can occur in publications in several ways. One is the omission of certain aspects or information, which can occur for various reasons, such as the intentional non-reporting of assumptions or non-significant results. However, the omission or neglect of relevant issues due to a lack of critical questioning of current guidelines or general practice can also lead to incomplete assessments and thus to bias in publications and the resulting meta-analysis.

With regard to this review, as a quantitative assessment of occupational oral exposure is not yet required, e.g., under REACH, oral exposure was not likely to be considered as a potentially contributing route in many publications. In particular, workplaces where oral exposure is not of concern according to the current state of knowledge, were not further investigated for this exposure pathway. According to this assumption, many publications that would conclude irrelevance could not be found with the applied search strategies because they did not mention the oral exposure pathway. Furthermore, the number of publications which concluded irrelevance may not reflect all associated workplaces, especially since the current standard is not to quantify occupational oral exposure.

This might explain the distribution of included publications, as the number of studies on irrelevance (*n* = 9) was smaller than the number of studies on potential relevance (*n* = 123) or relevance (*n* = 80) which might therefor be a consequence of bias. However, there may be other cases where occupational oral exposure is not relevant due to adherence to good hygiene, irrelevance of oral exposure due to specific workplaces or other yet unidentified reasons. Therefore, there is a risk of bias, in particular publication bias.

## 4 Discussion

Since the overall exposure of workers needs to be considered for a comprehensive assessment of worker exposure, this systematic review focused on the potential relevance of occupational oral exposure as a route of exposure that has not been the focus of research in the past.

In the course of the review, 147 publications were identified that contained conclusions on the relevance of oral exposure in the workplace. When examining the detailed categories in this review, both conclusions on the relevance and the irrelevance of occupational oral exposure were identified. However, the publications concluding a (potential) relevance of occupational oral exposure outnumbered those concluding no relevance. In particular, studies in which occupational oral exposure was deemed irrelevant might not mention this route of exposure in the resulting publications.

Still, the results of this review indicate that oral exposure may contribute significantly to the overall occupational exposure. According to the results of this review, this depends both on the workplaces and activities. In addition, the dependency on different substance groups was also investigated in this review. However, the database did not allow the identification of relevant or irrelevant substance groups here. Instead, it was only possible to identify groups of substances that have been more frequently focused on in the past.

The review also summarized the underlying modeling and measurement approaches. The most common modeling approaches included a constant ingestion rate, contact frequencies, or descriptions of the transfer. Measurements mostly investigated surface or air contaminations and biomonitoring. This shows that there are several different non-standardized approaches to occupational oral exposure assessment that are complex in their data collection requirements and are therefore complex in use.

### 4.1 Limitations and strengths

Due to the extensive search strings and the resulting number of publications identified, this systematic literature review was limited by the exclusion of studies published before the year 2000 and by the limitation of study inclusion by publication language (English and German). In addition, publications were only assessed by one author during the title and abstract and full text screening. However, the consistency between the authors and within the authors was improved by the consistency checks and the following discussion of criteria and assessments. In addition, the data extraction was not validated by a second author. Instead, the data extraction was performed in small steps starting with a qualitative extraction of relevant information into course categories to ensure a minimization of errors.

A strength of this systematic literature review was its differentiated search strategies, which included different study designs and strategies for drawing conclusions about oral exposure in the workplace, including direct statements as well as conclusions drawn from measurement or modeling approaches. In addition, the search not only included five databases but also websites of research institutes that can provide research reports, which are not covered by databases resulting in a comprehensive enhancement of the knowledge on the relevance of occupational oral exposure to date. Moreover, the sorting of records according to keywords allowed a comprehensive screening process and the identification of the most relevant publications from a large initial dataset. Finally, the developed concept of detailed categories in the concept matrix allowed a detailed and interpretable reflection of the current state of knowledge regarding the relevance of occupational oral exposure.

## 5 Conclusion

To the authors’ knowledge, this is the first systematic literature review on the relevance of oral exposure (to chemicals) in the workplace. It showed that occupational oral exposure can be relevant. So far, this has been documented mainly for other indoor workplaces, other industrial workplaces or recycling. However, an analogous identification of relevant substance groups is not yet possible due to a limited database, especially for liquids. Recent research has focused on the substance groups of metals, dusts and powders, and pesticides.

In order to comprehensively protect workers in terms of their overall exposure, the next step is to specify the conditions of its occurrence with respect to workplaces and substance groups, in particular liquids. Since current approaches to modeling and measurement of occupational oral exposure require complex information and considerable effort, simplified and standardized modeling and measurement approaches are needed for the future assessment of occupational oral exposure.

## Data availability statement

The original contributions presented in the study are included in the article/Supplementary material, further inquiries can be directed to the corresponding author.

## Author contributions

MD: Conceptualization, Formal analysis, Investigation, Methodology, Visualization, Writing – original draft. WS: Formal analysis, Investigation, Writing – review & editing. US: Conceptualization, Supervision, Writing – review & editing. AK: Conceptualization, Supervision, Writing – review & editing.
