# Supplementary material for: The relevance of oral exposure in the workplace: a systematic review and meta-analysis
Source: Front Public Health. 2023 Nov 30;11:1298744. doi: 10.3389/fpubh.2023.1298744 (PMC10720623; doi:10.3389/fpubh.2023.1298744)
Supplement: Supplementary file 1 [file Data_Sheet_1.pdf]

Supplementary Material

Supplementary Table 1: Overview of publications used for the evaluation of search strategies.

No	First author	Title	Year	Study
1	Cherrie	How important is inadvertent ingestion of hazardous substances at work	2006	(1)
2	Christopher	Inadvertent ingestion exposure in the workplace	2007	(2)
3	Deubner	Contribution of Incidental Exposure Pathways to Total Beryllium Exposures	2001	(3)
4	Hwang	Using structural equation model to explore occupational lead exposure pathways	2002	(4)
5	Gorman Ng	The Relationship between inadvertent ingestion and dermal exposure pathways	2012	(5)
6	Gorman Ng	Field Measurements of Inadvertent Ingestion Exposure to Metals	2017	(6)
7	Christopher	Inadvertent ingestion exposure to hazardous substances in the workplace	2008	(7)
8	Sinclair	Measuring water ingestion from spray exposures	2016	(8)
9	Beattie	The use of bio monitoring to assess exposure in the electroplating industry	2017	(9)
10	Phanprasit	Inhalation and dermal exposure to toluene among printing workers in a plastic bag factory	2019	(10)

11	Viegas	HBM4EU Chromates Study Determinants of Exposure to Hexavalent Chromium in Plating Welding and Other Occupational Settings	2022	(11)
12	Verdonck	Systematic review of biomonitoring data on occupational exposure to hexavalent chromium	2021	(12)
13	Keen	Exposure to hexavalent chromium, nickel and cadmium compounds in the electroplating industry	2013	(13)
14	Connolly	Evaluating Glyphosate Exposure Routes and Their Contribution to Total Body Burden A Study Among Amenity Horticulturalists	2019	(14)
15	Hwang	Monitoring of arsenic exposure with speciated urinary inorganic arsenic metabolites for ion implanter maintenance engineers	2002	(15)

Supplementary Table 2: Evaluation results for search strategies. Numbers of publications refer to Supplementary Table 1. Yes (Y) and No (N) statements inform on whether the publication is under the search results for the respective search strategy and database. Dates of search are 10/28/2022 for Web of Science and 10/31/2022 for PubMed.

**Database**	**Web of Science**								**PubMed**							
**Search strategy** / **No**	**Availability**	**1**	**2**	**3**	**4**	**5**	**6**	**7**	**Availability**	**1**	**2**	**3**	**4**	**5**	**6**	**7**
1	Y	Y	Y	N	N	N	N	Y	Y	Y	Y	N	N	N	N	Y
2	N	-	-	-	-	-	-	-	N	-	-	-	-	-	-	-
3	N	-	-	-	-	-	-	-	Y	Y	N	N	N	N	N	N

4	Y	Y	Y	N	N	N	N	N	Y	Y	Y	N	N	N	N	N
5	Y	Y	Y	N	N	N	N	N	Y	Y	Y	N	N	N	N	N
6	Y	Y	Y	N	N	N	N	N	Y	Y	Y	N	N	N	N	N
7	N	-	-	-	-	-	-	-	N	-	-	-	-	-	-	-
8	Y	N	N	N	N	N	N	Y	Y	Y	Y	N	Y	Y	N	N
9	Y	Y	Y	Y	N	N	N	N	Y	Y	Y	Y	N	N	N	N
10	N	-	-	-	-	-	-	-	N	-	-	-	-	-	-	-
11	N	-	-	-	-	-	-	-	Y	N	N	Y	N	N	N	N
12	Y	N	N	Y	N	N	N	N	Y	N	N	Y	N	N	N	N
13	N	-	-	-	-	-	-	-	N	-	-	-	-	-	-	
14	Y	N	Y	Y	N	N	N	Y	Y	N	Y	Y	N	N	N	Y
15	N	-	-	-	-	-	-	-	Y	N	N	N	N	N	N	N

Supplementary Table 3: Dates of last search for all search strategies, databases and websites. Simplified search strategies for websites.

**Search strategy**	**Database / Website**	**Date of last search**
1	WebOfScience	11/16/.2022
2	WebOfScience	11/17/2022

3	WebOfScience	11/17/2022
4	WebOfScience	11/17/2022
5	WebOfScience	11/17/2022
6	WebOfScience	11/18/2022
7	WebOfScience	11/18/2022
1	PubMed	11/14/2022
2	PubMed	11/17/2022
3	PubMed	11/17/2022
4	PubMed	11/17/2022
5	PubMed	11/17/2022
6	PubMed	11/18/2022
7	PubMed	11/18/2022
1-7	Cochrane	02/09/2023
1-7	Bergischbib	02/14/2023
1-7	Deutsche Nationalbibliothek	02/14/2023
Occupational ingestion	IOM	11/22/2022
Occupational oral Exposure	IOM	11/23/2022

Occupational oral intake	IOM	11/22/2022
Occupational oral Exposure	HSE	11/25/2022
Occupational ingestion	HSE	11/24/2022
Occupational oral intake	HSE	11/24-25/2022
Occupational oral Exposure	BAuA	11/23/2022
Oral exposure workplace	BAuA	11/23/2022
Oral exposure worker	BAuA	11/23/2022
ingestion	RIVM	11/22/2022
oral	RIVM	11/23/2022
intake	RIVM	11/23/2022
Occupational ingestion	TNO	11/22/2022
Occupational oral exposure	TNO	11/22/2022
Occupational AND oral AND exposure	EPA	11/28-29/2022
Occupational AND ingestion	EPA	11/28-29/2022
Occupational AND oral AND exposure	NIOSH	11/29/2022
Occupational AND ingestion	NIOSH	11/29/2022
Occupational AND ingestion	OECD	11/30/2022

Occupational AND oral AND exposure	OECD	11/30/2022

Supplementary Table 4: Overview of all 147 included studies with extracted information for the work sector, the substance group and the categorization of irrelevance, potential relevance and relevance.

First author	Year	Study	Work sector	Substance group	Irrele vant	Pot. relevant	Rele vant
Beattie	2017	(9)	Metalworking	Metals		x	x
Caballero-Casero	2022	(16)	Agriculture	Dust / powder		x	x
Cherrie	2006	(1)	n.a.	Metals, High molecular weight allergens, Pharmaceuticals, Pesticides, Biological substances / pathogens / microorganisms, Radioisotopes / radiation		x	x
Choy	2004	(17)	Other industry	Metals		x	x
Ciani	2021	(18)	Indoor	Dust / powder		x	x
Abebe Debela	2021	(19)	Recycling (e-waste)	Soil		x	x
Deubner	2001	(3)	n.a.	Metals		x	
Geraldino	2021	(20)	Outdoor	Organic compounds			x

Gorce	2015	(21)	Accumulators	Metals		x	
Gorman Ng	2017	(6)	Metalworking	Metals		x	
Gorman Ng	2012	(5)	n.a.	Dust / powder, Organic compounds, Biological substances / pathogens / microorganisms, Radioisotopes / radiation		x	
Hubbard	2022	(22)	n.a.	Dust / powder, Soil, Biological substances / pathogens / microorganisms		x	
Hwang	2002	(4)	Accumulators	Metals		x	x
Hwang	2000	(23)	Accumulators	Metals		x	x
Hwang	2000	(24)	Laboratory	Metals		x	
Julian	2018	(25)	Agriculture	Biological substances / pathogens / microorganisms		x	
Lupolt	2022	(26)	Agriculture	Soil		x	x
Nduka	2019	(27)	Workshop	Metals, Dust / powder		x	
Sen	2002	(28)	Construction	Metals, Dust / powder		x	x
Sinclair	2016	(8)	Indoor	(Waste) Water		x	

Tao	2015	(29)	Recycling (e-waste)	Metals,Dust / powder		x	
Tran	2022	(30)	Indoor, Other industry	Dust / powder		x	x
Xing	2011	(31)	Recycling (e-waste)	Dust / powder		x	
Alsohaimi	2020	(32)	Outdoor	Metals, Dust / powder		x	x
Chou	2016	(33)	Indoor, Other industry	Dust / powder		x	x
Hamilton	2004	(34)	Other industry	Dust / powder, Radioisotopes / radiation		x	
Huang	2017	(35)	Construction	Metals, Organic compounds, Pesticides		x	x
Julander	2020	(36)	Metalworking	Metals		x	x
Li	2016	(37)	Waste management	Dust / powder, Soil, Organic compounds, Food		x	x
Liu	2013	(38)	Indoor	Dust / powder		x	
Nduka	2016	(39)	Workshop	Metals, Dust / powder		x	
Ohajinwa	2019	(40)	Recycling (e-waste)	Metals		x	x

Sklar	2021	(41)	Nursing	Biological substances / pathogens / microorganisms		x	
Su	2022	(42)	Outdoor, Indoor	Dust / powder		x	x
Zhang	2021	(43)	Recycling (e-waste)	Dust / powder		x	x
Zhao	2022	(44)	Recycling (e-waste)	Dust / powder		x	
Connolly	2019	(14)	Pest control	Pesticides		x	
Dor	2003	(45)	Energy	Soil		x	
Ellis	2018	(46)	Weapons	Dust / powder, Radioisotopes / radiation	x		x
Folarin	2022	(47)	Energy	Soil		x	
Gorman Ng	2011	(48)	Pest control	Pesticides		x	
Gwenzi	2020	(49)	Indoor	Biological substances / pathogens / microorganisms		x	x
Harrison	2003	(50)	n.a.	Radioisotopes / radiation		x	
Jandard	2018	(51)	n.a.	Pharmaceuticals		x	

Kademoglou	2017	(52)	Indoor	Dust / powder, Flame retardants		x	
Klous	2016	(53)	Agriculture	Biological substances / pathogens / microorganisms		x	
Nguyen	2019	(54)	Recycling (e-waste)	Dust / powder, Flame retardants		x	
Obiri	2016	(55)	Recycling (e-waste)	Metals, Dust / powder, Soil		x	
Obiri	2016	(56)	Outdoor	Metals			x
Panagiotou	2022	(57)	Agriculture	(Waste) Water, Soil, Biological substances / pathogens / microorganisms			x
Pendergrass	2000	(58)	Pest control	Pesticides		x	
Rocha	2021	(59)	Nursing	Pharmaceuticals		x	x
Semerjian	2018	(60)	Agriculture	Pharmaceuticals, (Waste) Water		x	
Shankar	2017	(61)	Polymerss	Organic compounds		x	
Stewart	2009	(62)	Laboratory	Biological substances / pathogens / microorganisms	x	x	x

Szrom	2009	(63)	Weapons	Radioisotopes / radiation		x	
Xu	2016	(64)	n.a.	Dust / powder, Flame retardants			x
Zhang	2021	(65)	Metalworking	Metals		x	
Beyer	2014	(66)	Workshop	Metals	x	x	x
Chang	2003	(67)	Laboratory	Others	x	x	x
Cote	2018	(68)	Pest control	Pesticides		x	x
da Cunha	2002	(69)	Other industry	Dust / powder		x	x
Enander	2004	(70)	Workshop	Metals, Dust / powder		x	x
Fernandez -Landero	2021	(71)	Agriculture	Metals, Soil		x	x
Iwegbue	2020	(72)	Workshop	Metals, Dust / powder		x	x
Lau	2014	(73)	Workshop, Recycling (e-waste)	Metals, Dust / powder		x	x
Gorman Ng	2016	(74)	n.a.	Others		x	
Protano	2022	(75)	n.a.	Others		x	
Zhu	2021	(76)	Waste management	Soil		x	x

Bousoumah	2021	(77)	Polymerss	Organic compounds		x	
Bühl	2017	(78)	Other industry	Metals		x	
Connolly	2018	(79)	Pest control	Pesticides		x	
Dai	2020	(80)	Recycling (e-waste)	Organic compounds			x
Fong	2014	(81)	Polymerss	Plasticizers	x	x	
Fréry	2020	(82)	Polymerss	Plasticizers		x	
Galea	2021	(83)	Metalworking	Metals			x
Kapka-Skrzypczak	2011	(84)	Pest control	Pesticides	x	x	
Koh	2020	(85)	n.a.	Organic compounds		x	
Leso	2022	(86)	Nursing	Pharmaceuticals		x	
Ndaw	2021	(87)	Agriculture	Dust / powder		x	
Santonen	2019	(88)	Metalworking	Metals		x	
Santonen	2022	(89)	Metalworking, Other industry	Metals		x	
Talaska	2014	(90)	Other industry	Organic compounds		x	
Tavares	2022	(91)	Metalworking	Metals			x

Vandebroek	2019	(92)	Weapons	Metals		x	
Verdonck	2021	(12)	Metalworking, Other industry	Metals		x	
Viegas	2022	(11)	Metalworking	Metals		x	x
Wittsiepe	2017	(93)	Recycling (e-waste)	Metals		x	
Ferland	2015	(94)	Pest control	Pesticides		x	
Hardt	2003	(95)	Pest control	Pesticides		x	
Heinala	2017	(96)	Other industry	Dust / powder		x	
Ndaw	2022	(97)	Metalworking	Metals		x	
Schecter	2009	(98)	Recycling (e-waste)	Dust / powder		x	
Scheepers	2021	(99)	Recycling (e-waste)	Metals, Flame retardants, Plasticizers		x	
Aribou	2022	(100)	Pest control	Pesticides			x
Jaga	2006	(101)	Pest control	Pesticides			x
Pedroso	2022	(102)	Pest control	Pesticides		x	
Thompson	2021	(103)	Nursing	Pharmaceuticals		x	

WHO	2001	(104)	Weapons	Dust / powder		x	
Georgia Department of Public Health	2017	(105)	Recycling (e-waste), Other industry, Weapons	Metals			x
Jackson	2018	(106)	Weapons	Metals		x	
Hughson	2005	(107)	Other industry	Metals		x	
Sewell	2006	(108)	Pest control	Pesticides		x	
Aitken	2004	(109)	Other industry	Nanoparticles		x	
Christopher	2007	(2)	n.a.	Metals, Biological substances / pathogens / microorganisms, High molecular weight allergens, Pharmaceuticals, Pesticides, Radioisotopes / radiation		x	x
Allaouat	2020	(110)	n.a.	Metals			x
Burton	2020	(111)	Nursing	Biological substances / pathogens / microorganisms		x	
Amoah	2022	(112)	Waste management	Biological substances / pathogens / microorganisms, (Waste) Water		x	x

Bakhmutsky	2011	(113)	Weapons	Radioisotopes / radiation		x	x
Chen	2019	(114)	Indoor, Recycling (e-waste), Other industry, Workshop	Dust / powder, Flame retardants		x	x
Damian	2011	(115)	Energy	Metals	x	x	x
Fang	2013	(116)	Recycling (e-waste)	Metals, Dust / powder			x
Flack	2012	(117)	Metalworking, Pest control	Metals, Pesticides		x	
Guney	2016	(118)	n.a.	Dust / powder, Plasticizers		x	x
Hu	2020	(119)	Other industry	Metals		x	x
Li	2015	(120)	Indoor	Dust / powder, Flame retardants		x	x
Perry	2006	(121)	Agriculture, Pest control	Pesticides	x		
Qin	2021	(122)	Recycling (e-waste)	Dust / powder, Flame retardants		x	x
Qu	2012	(123)	Outdoor, Indoor, Other industry	Metals		x	x
Ritchie	2001	(124)	Energy	Organic compounds	x	x	x

Roldan-Tapia	2005	(125)	Agriculture	Pesticides			x
Waheed	2017	(126)	Other industry	Dust / powder, Pesticides			x
Barker	2017	(127)	Indoor	(Waste) Water, Biological substances / pathogens / microorganisms		x	
Chiang	2009	(128)	Indoor	Organic compounds			x
Gulson	2005	(129)	Outdoor	Metals, Dust / powder, Radioisotopes / radiation		x	
Jones-Otazo	2005	(130)	Recycling (e-waste)	Dust / powder, Flame retardants			x
Kamal	2014	(131)	Other industry	Dust / powder, Organic compounds		x	x
Krzyzanoswki	2016	(132)	Agriculture	(Waste) Water, Biological substances / pathogens / microorganisms		x	x
Li	2022	(133)	Recycling (e-waste)	Dust / powder, Flame retardants			x
Liu	2018	(134)	Indoor	Organic compounds		x	x
Liu	2018	(135)	Outdoor, Indoor	Dust / powder, Flame retardants		x	x

Lurker	2014	(136)	Pest control, Weapons	Dust / powder, Pesticides			x
Othman	2018	(137)	Indoor	Metals, Dust / powder			x
Ali	2017	(138)	Workshop	Dust / powder, Organic compounds		x	x
Fernandez-Caliani	2019	(139)	Agriculture, Other industry	Metals, Flame retardants		x	x
Zou	2020	(140)	Energy	Metals, Soil		x	x
Xia	2018	(141)	Indoor	Organic compounds			x
Zhang	2019	(142)	Recycling (e-waste)	Organic compounds, Flame retardants			x
Zou	2021	(143)	Energy	Organic compounds, Soil		x	x
Guo	2019	(144)	Other industry	Organic compounds		x	x
Shen	2019	(145)	Recycling (e-waste)	Dust / powder, Flame retardants		x	x
Wannomai	2020	(146)	Recycling (e-waste)	Dust / powder, Flame retardants		x	x
Shen	2022	(147)	Energy	Metals, Dust / powder		x	x
Gao	2015	(148)	Indoor	Metals, Dust / powder		x	x
Gerritsen-Ebben	2007	(149)	Agriculture	Pesticides		x	

Die	2019	(150)	Recycling (e-waste)	Dust / powder, Flame retardants			x
Zhou	2014	(151)	Other industry	Dust / powder, Flame retardants, (Waste) Water		x	x

References

1. Cherrie JW, Semple S, Christopher Y, Saleem A, Hughson GW, Philips A. How important is inadvertent ingestion of hazardous substances at work? Ann Occup Hyg. 2006;50(7):693-704.

2. Y Christopher SSGWH, Cherrie JW. Inadvertent ingestion exposure in. 2007.

3. Deubner DC, Lowney YW, Paustenbach DJ, Warmerdam J. Contribution of incidental exposure pathways to total beryllium exposures. Appl Occup Environ Hyg. 2001;16(5):568-78.

4. Hwang YH, Chang CW, Chao KY, Hsiao FT, Chang HL, Han HZ. Using structural equation model to explore occupational lead exposure pathways. Sci Total Environ. 2002;284(1-3):95-108.

5. Gorman Ng M, Semple S, Cherrie JW, Christopher Y, Northage C, Tielemans E, et al. The relationship between inadvertent ingestion and dermal exposure pathways: a new integrated conceptual model and a database of dermal and oral transfer efficiencies. Ann Occup Hyg. 2012;56(9):1000-12.

6. Gorman Ng M, MacCalman L, Semple S, van Tongeren M. Field Measurements of Inadvertent Ingestion Exposure to Metals. Ann Work Expo Health. 2017;61(9):1097-107.

7. Christopher Y. Inadvertent ingestion exposure to hazardous substances in the workplace: University of Aberdeen; 2008.

8. Sinclair M, Roddick F, Nguyen T, O'Toole J, Leder K. Measuring water ingestion from spray exposures. Water Res. 2016;99:1-6.

9. Beattie H, Keen C, Coldwell M, Tan E, Morton J, McAlinden J, et al. The use of bio-monitoring to assess exposure in the electroplating industry. J Expo Sci Environ Epidemiol. 2017;27(1):47-55.

10. Phanprasit W, Songpek K, Boonyayothin V, Sujirarat D. Inhalation and dermal exposure to toluene among printing workers in a plastic bag factory. Journal of Health Research. 2019;33(1):68-79.

11. Viegas S, Martins C, Bocca B, Bousoumah R, Duca RC, Galea KS, et al. HBM4EU Chromates Study: Determinants of Exposure to Hexavalent Chromium in Plating, Welding and Other Occupational Settings. Int J Environ Res Public Health. 2022;19(6).

12. Verdonck J, Duca RC, Galea KS, Iavicoli I, Poels K, Töreyin ZN, et al. Systematic review of biomonitoring data on occupational exposure to hexavalent chromium. Int J Hyg Environ Health. 2021;236:113799.

13. Keen C, Tan E, McAlinden J, Woolgar P, Smith P. Exposure to hexavalent chromium, nickel and cadmium compounds in the electroplating industry. Health and Safety Executive; 2013.

14. Connolly A, Coggins MA, Galea KS, Jones K, Kenny L, McGowan P, et al. Evaluating Glyphosate Exposure Routes and Their Contribution to Total Body Burden: A Study Among Amenity Horticulturalists. Ann Work Expo Health. 2019;63(2):133-47.

15. Hwang Y-H, Lee Z-Y, Wang J-D, Hsueh Y-M, Lu IC, Yao W-L. Monitoring of arsenic exposure with speciated urinary inorganic arsenic metabolites for ion implanter maintenance engineers. Environ Res. 2002;90(3):207-16.

16. Caballero-Casero N, Rubio S. Identification of bisphenols and derivatives in greenhouse dust as a potential source for human occupational exposure. Anal Bioanal Chem. 2022;414(18):5397-409.

17. Choy KD, Lee HS, Tan CH. Blood lead monitoring in a decorative ceramic tiles factory in Singapore. Singapore Med J. 2004;45(4):176-9.

18. Ciani F, Chiarantini L, Costagliola P, Rimondi V. Particle-Bound Mercury Characterization in the Central Italian Herbarium of the Natural History Museum of the University of Florence (Italy). Toxics. 2021;9(6).

19. Debela SA, Sheriff I, Debela EA, Sesay MT, Tolcha A, Tengbe MS. Assessment of Perceptions and Cancer Risks of Workers at a Polychlorinated Biphenyl-Contaminated Hotspot in Ethiopia. J Health Pollut. 2021;11(30):210609.

20. Geraldino BR, Nunes RFN, Gomes JB, da Poça KS, Toledo TMP, Otero UB, et al. Risks related to the domestic laundering of filling station attendant uniforms: advances and uncertainties. Rev Bras Med Trab. 2021;19(2):240-8.

21. Gorce JP, Roff M. Hand Self-Wiping Protocol for the Investigation of Lead Exposure in the Workplace. J Occup Environ Hyg. 2015;12(10):699-707.

22. Hubbard H, Özkaynak H, Glen G, Cohen J, Thomas K, Phillips L, et al. Model-based predictions of soil and dust ingestion rates for U.S. adults using the stochastic human exposure and dose simulation soil and dust model. Sci Total Environ. 2022;846:157501.

23. Hwang YH, Chao KY, Chang CW, Hsiao FT, Chang HL, Han HZ. Lip lead as an alternative measure for lead exposure assessment of lead battery assembly workers. Aihaj. 2000;61(6):825-31.

24. Hwang YH, Chen SC. Monitoring of low level arsenic exposure during maintenance of ion implanters. Arch Environ Health. 2000;55(5):347-54.

25. Julian TR, Vithanage HSK, Chua ML, Kuroda M, Pitol AK, Nguyen PHL, et al. High time-resolution simulation of E. coli on hands reveals large variation in microbial exposures amongst Vietnamese farmers using human excreta for agriculture. Sci Total Environ. 2018;635:120-31.

26. Lupolt SN, Agnew J, Ramachandran G, Burke TA, Kennedy RD, Nachman KE. A qualitative characterization of meso-activity factors to estimate soil exposure for agricultural workers. J Expo Sci Environ Epidemiol. 2022.

27. Nduka JK, Kelle HI, Amuka JO. Health risk assessment of cadmium, chromium and nickel from car paint dust from used automobiles at auto-panel workshops in Nigeria. Toxicol Rep. 2019;6:449-56.

28. Sen D, Wolfson H, Dilworth M. Lead exposure in scaffolders during refurbishment construction activity--an observational study. Occup Med (Lond). 2002;52(1):49-54.

29. Tao XQ, Shen DS, Shentu JL, Long YY, Feng YJ, Shen CC. Bioaccessibility and health risk of heavy metals in ash from the incineration of different e-waste residues. Environ Sci Pollut Res Int. 2015;22(5):3558-69.

30. Tran LT, Kieu TC, Bui HM, Nguyen NT, Nguyen TTT, Nguyen DT, et al. Polybrominated diphenyl ethers in indoor dusts from industrial factories, offices, and houses in northern Vietnam: Contamination characteristics and human exposure. Environ Geochem Health. 2022;44(8):2375-88.

31. Xing GH, Liang Y, Chen LX, Wu SC, Wong MH. Exposure to PCBs, through inhalation, dermal contact and dust ingestion at Taizhou, China--a major site for recycling transformers. Chemosphere. 2011;83(4):605-11.

32. Alsohaimi IH, El-Hashemy MA, Al-Ruwaili AG, Seaf El-Nasr TA, Almuaikel NS. Assessment of Trace Elements in Urban Road Dust of a City in a Border Province Concerning Their Levels, Sources, and Related Health Risks. Arch Environ Contam Toxicol. 2020;79(1):23-38.

33. Chou HM, Kao CC, Chuang KP, Lin C, Shy CG, Chen RF, et al. Levels of Polybrominated Diphenyl Ethers in Air-Conditioner Filter Dust Used to Assess Health Risks in Clinic and Electronic Plant Employees. Aerosol Air Qual Res. 2016;16(1):184-94.

34. Hamilton IS, Arno MG, Rock JC, Berry RO, Poston JW, Sr., Cezeaux JR, et al. Radiological assessment of petroleum pipe scale from pipe-rattling operations. Health Phys. 2004;87(4):382-97.

35. Huang S, Zhao X, Sun YQ, Ma JL, Gao XF, Xie T, et al. Pollution of hazardous substances in industrial construction and demolition wastes and their multi-path risk within an abandoned pesticide manufacturing plant. Front Env Sci Eng. 2017;11(1):13.

36. Julander A, Midander K, Garcia-Garcia S, Vihlborg P, Graff P. A Case Study of Brass Foundry Workers' Estimated Lead (Pb) Body Burden from Different Exposure Routes. Ann Work Expos Health. 2020;64(9):970-81.

37. Li JF, Dong H, Sun J, Tang JS, Nie JH, Zhang SY, et al. Composition profiles, monthly changes and health risk of PCDD/F in fly ash discharged from a municipal solid waste incinerator (MSWI) in Northeast China. RSC Adv. 2016;6(113):111966-75.

38. Liu NN, Shi YL, Xu L, Li WH, Cai YQ. Occupational exposure to synthetic musks in barbershops, compared with the common exposure in the dormitories and households. Chemosphere. 2013;93(9):1804-10.

39. Nduka JK, Amuka JPO, Onwuka JC, Udowelle NA, Orisakwe OE. Human health risk assessment of lead, manganese and copper from scrapped car paint dust from automobile workshops in Nigeria. Environ Sci Pollut Res. 2016;23(20):20341-9.

40. Ohajinwa CM, van Bodegom PM, Osibanjo O, Xie Q, Chen JW, Vijver MG, et al. Health Risks of Polybrominated Diphenyl Ethers (PBDEs) and Metals at Informal Electronic Waste Recycling Sites. Int J Environ Res Public Health. 2019;16(6):19.

41. Sklar R, Zhou ZY, Ndayisaba W, Muspratt A, Fuhrmeister ER, Nelson K, et al. Risk of adenovirus and Cryptosporidium ingestion to sanitation workers in a municipal scale non-sewered sanitation process: a case study from Kigali, Rwanda. J Wate Sanit Hyg Dev. 2021;11(4):570-8.

42. Su CK, Lu JH, Chao HR, Chang WH, Tsai MH, Wang CL, et al. Polybrominated Dibenzo-p-dioxins/Furans (PBDD/Fs) and Diphenyl Ethers (PBDEs) in the Indoor and Outdoor of Gymnasiums. Aerosol Air Qual Res. 2022;22(9):16.

43. Zhang QY, Li XJ, Wang Y, Zhang C, Cheng ZP, Zhao LC, et al. Occurrence of novel organophosphate esters derived from organophosphite antioxidants in an e-waste dismantling area: Associations between hand wipes and dust. Environ Int. 2021;157:8.

44. Zhao LC, Lu Y, Zhu HK, Cheng ZP, Wang Y, Chen H, et al. E-waste dismantling-related occupational and routine exposure to melamine and its derivatives: Estimating exposure via dust ingestion and hand-to-mouth contact. Environ Int. 2022;165:10.

45. Dor F, Empereur-Bissonnet P, Zmirou D, Nedellec V, Haguenoer JM, Jongeneelen F, et al. Validation of multimedia models assessing exposure to PAHs--the SOLEX study. Risk Anal. 2003;23(5):1047-57.

46. Ellis ED, Boice JD, Jr., Golden AP, Girardi DJ, Cohen SS, Mumma MT, et al. Dosimetry is Key to Good Epidemiology: Workers at Mallinckrodt Chemical Works had Seven Different Source Exposures. Health Phys. 2018;114(4):386-97.

47. Folarin BT, Abdallah M, Oluseyi TO, Harrad S, Olayinka KO. Concentrations and Toxic Implications of Dioxin-Like Polychlorinated Biphenyls in Soil Samples from Electrical Power Stations in Lagos, Nigeria. Environ Toxicol Chem. 2022;41(3):800-9.

48. Gorman Ng M, Stjernberg E, Koehoorn M, Demers PA, Davies HW. Exposure to pesticides and metal contaminants of fertilizer among tree planters. Ann Occup Hyg. 2011;55(7):752-63.

49. Gwenzi W. The 'thanato-resistome' - The funeral industry as a potential reservoir of antibiotic resistance: Early insights and perspectives. Sci Total Environ. 2020;749:141120.

50. Harrison JD, Muirhead CR. Quantitative comparisons of cancer induction in humans by internally deposited radionuclides and external radiation. Int J Radiat Biol. 2003;79(1):1-13.

51. Jandard C, Hemming H, Prause M, Sehner C, Schwind M, Abromovitz M, et al. Applicability of surface sampling and calculation of surface limits for pharmaceutical drug substances for occupational health purposes. Regul Toxicol Pharmacol. 2018;95:434-41.

52. Kademoglou K, Xu F, Padilla-Sanchez JA, Haug LS, Covaci A, Collins CD. Legacy and alternative flame retardants in Norwegian and UK indoor environment: Implications of human exposure via dust ingestion. Environ Int. 2017;102:48-56.

53. Klous G, Huss A, Heederik DJJ, Coutinho RA. Human-livestock contacts and their relationship to transmission of zoonotic pathogens, a systematic review of literature. One Health. 2016;2:65-76.

54. Nguyen LV, Diamond ML, Venier M, Stubbings WA, Romanak K, Bajard L, et al. Exposure of Canadian electronic waste dismantlers to flame retardants. Environ Int. 2019;129:95-104.

55. Obiri S, Ansa-Asare OD, Mohammed S, Darko HF, Dartey AG. Exposure to toxicants in soil and bottom ash deposits in Agbogbloshie, Ghana: human health risk assessment. Environ Monit Assess. 2016;188(10):583.

56. Obiri S, Yeboah PO, Osae S, Adu-Kumi S, Cobbina SJ, Armah FA, et al. Human Health Risk Assessment of Artisanal Miners Exposed to Toxic Chemicals in Water and Sediments in the Prestea Huni Valley District of Ghana. Int J Environ Res Public Health. 2016;13(1).

57. Panagiotou CF, Stefan C, Papanastasiou P, Sprenger C. Quantitative microbial risk assessment (QMRA) for setting health-based performance targets during soil aquifer treatment. Environ Sci Pollut Res Int. 2022.

58. Pendergrass SM, Krake AM, Jaycox LB. Development of a versatile method for the detection of nicotine in air. Aihaj. 2000;61(4):469-72.

59. Rocha SD, Gomes ANH, Zen PRG, Bica CG. Handling of antineoplastic drugs: a health concern among health care workers. Rev Bras Med Trab. 2021;18(4):407-14.

60. Semerjian L, Shanableh A, Semreen MH, Samarai M. Human health risk assessment of pharmaceuticals in treated wastewater reused for non-potable applications in Sharjah, United Arab Emirates. Environ Int. 2018;121(Pt 1):325-31.

61. Shankar K, Fung V, Seneviratne M, O'Donnell GE. Exposure to 4,4'-methylene bis (2-chloroaniline) (MbOCA) in New South Wales, Australia. J Occup Health. 2017;59(3):296-303.

62. Stewart I, Carmichael WW, Sadler R, McGregor GB, Reardon K, Eaglesham GK, et al. Occupational and environmental hazard assessments for the isolation, purification and toxicity testing of cyanobacterial toxins. Environ Health. 2009;8:52.

63. Szrom F, Falo GA, Lodde GM, Parkhurst MA, Daxon EG. Inhalation and ingestion intakes with associated dose estimates for level II and level III personnel using Capstone study data. Health Phys. 2009;96(3):363-79.

64. Xu F, Tang W, Zhang W, Liu L, Lin K. Levels, distributions and correlations of polybrominated diphenyl ethers in air and dust of household and workplace in Shanghai, China: implication for daily human exposure. Environ Sci Pollut Res Int. 2016;23(4):3229-38.

65. Zhang L, Zhou H, Chen X, Liu G, Jiang C, Zheng L. Study of the micromorphology and health risks of arsenic in copper smelting slag tailings for safe resource utilization. Ecotoxicol Environ Saf. 2021;219:112321.

66. Beyer LA, Greenberg G, Beck BD. Evaluation of Potential Exposure to Metals in Laundered Shop Towels. Hum Ecol Risk Assess. 2014;20(1):111-36.

67. Chang SJ, Lamm SH. Human health effects of sodium azide exposure: A literature review and analysis. Int J Toxicol. 2003;22(3):175-86.

68. Cote J, Bouchard M. Dose reconstruction in workers exposed to two major pyrethroid pesticides and determination of biological reference values using a toxicokinetic model. J Expo Sci Environ Epidemiol. 2018;28(6):599-614.

69. da Cunha KD, Lipsztein JL, Azeredo AM, Melo D, Juliao L, Lamego FF, et al. Study of worker's exposure to thorium, uranium and niobium mineral dust. Water Air Soil Pollut. 2002;137(1-4):45-61.

70. Enander RT, Cohen HJ, Gute DM, Brown LC, Desmaris AMC, Missaghian R. Lead and methylene chloride exposures among automotive repair technicians. J Occup Environ Hyg. 2004;1(2):119-25.

71. Fernandez-Landero S, Giraldez I, Fernandez-Caliani JC. Predicting the relative oral bioavailability of naturally occurring As, Cd and Pb from in vitro bioaccessibility measurement: implications for human soil ingestion exposure assessment. Environ Geochem Health. 2021;43(10):4251-64.

72. Iwegbue CMA, Nwose N, Egobueze FE, Odali EW, Tesi GO, Nwajei GE, et al. Risk assessment of human exposure to potentially toxic metals in indoor dust from some small and

medium scale enterprise workplace environments in southern Nigeria. Indoor Built Environ. 2020;29(8):1137-54.

73. Lau WKY, Liang P, Man YB, Chung SS, Wong MH. Human health risk assessment based on trace metals in suspended air particulates, surface dust, and floor dust from e-waste recycling workshops in Hong Kong, China. Environ Sci Pollut Res. 2014;21(5):3813-25.

74. Ng MG, Davis A, van Tongeren M, Cowie H, Semple S. Inadvertent ingestion exposure: hand- and object-to-mouth behavior among workers. J Expo Sci Environ Epidemiol. 2016;26(1):9-16.

75. Protano C, Buomprisco G, Cammalleri V, Pocino RN, Marotta D, Simonazzi S, et al. The Carcinogenic Effects of Formaldehyde Occupational Exposure: A Systematic Review. Cancers. 2022;14(1):12.

76. Zhu WH, Yang XT, He J, Wang XH, Lu R, Zhang Z. Investigation and Systematic Risk Assessment in a Typical Contaminated Site of Hazardous Waste Treatment and Disposal. Front Public Health. 2021;9:12.

77. Bousoumah R, Leso V, Iavicoli I, Huuskonen P, Viegas S, Porras SP, et al. Biomonitoring of occupational exposure to bisphenol A, bisphenol S and bisphenol F: A systematic review. Sci Total Environ. 2021;783:146905.

78. Bühl V, Álvarez MC, Torre MH, Pistón M, Mañay N. Biomonitoring of arsenic in woodworkers exposed to CCA and evaluation of other non-occupational sources in Uruguay. Int J Occup Environ Health. 2017;23(1):71-80.

79. Connolly A, Basinas I, Jones K, Galea KS, Kenny L, McGowan P, et al. Characterising glyphosate exposures among amenity horticulturists using multiple spot urine samples. Int J Hyg Environ Health. 2018;221(7):1012-22.

80. Dai Q, Xu X, Eskenazi B, Asante KA, Chen A, Fobil J, et al. Severe dioxin-like compound (DLC) contamination in e-waste recycling areas: An under-recognized threat to local health. Environ Int. 2020;139:105731.

81. Fong JP, Lee FJ, Lu IS, Uang SN, Lee CC. Estimating the contribution of inhalation exposure to di-2-ethylhexyl phthalate (DEHP) for PVC production workers, using personal air sampling and urinary metabolite monitoring. Int J Hyg Environ Health. 2014;217(1):102-9.

82. Fréry N, Santonen T, Porras SP, Fucic A, Leso V, Bousoumah R, et al. Biomonitoring of occupational exposure to phthalates: A systematic review. Int J Hyg Environ Health. 2020;229:113548.

83. Galea KS, Porras SP, Viegas S, Bocca B, Bousoumah R, Duca RC, et al. HBM4EU chromates study - Reflection and lessons learnt from designing and undertaking a collaborative European biomonitoring study on occupational exposure to hexavalent chromium. Int J Hyg Environ Health. 2021;234:113725.

84. Kapka-Skrzypczak L, Cyranka M, Skrzypczak M, Kruszewski M. Biomonitoring and biomarkers of organophosphate pesticides exposure - state of the art. Ann Agric Environ Med. 2011;18(2):294-303.

85. Koh DH, Park JH, Lee SG, Kim HC, Choi S, Jung H, et al. Comparison of Polycyclic Aromatic Hydrocarbons Exposure Across Occupations Using Urinary Metabolite 1-Hydroxypyrene. Ann Work Expo Health. 2020;64(4):445-54.

86. Leso V, Sottani C, Santocono C, Russo F, Grignani E, Iavicoli I. Exposure to Antineoplastic Drugs in Occupational Settings: A Systematic Review of Biological Monitoring Data. Int J Environ Res Public Health. 2022;19(6).

87. Ndaw S, Jargot D, Antoine G, Denis F, Melin S, Robert A. Investigating Multi-Mycotoxin Exposure in Occupational Settings: A Biomonitoring and Airborne Measurement Approach. Toxins (Basel). 2021;13(1).

88. Santonen T, Alimonti A, Bocca B, Duca RC, Galea KS, Godderis L, et al. Setting up a collaborative European human biological monitoring study on occupational exposure to hexavalent chromium. Environ Res. 2019;177:108583.

89. Santonen T, Porras SP, Bocca B, Bousoumah R, Duca RC, Galea KS, et al. HBM4EU chromates study - Overall results and recommendations for the biomonitoring of occupational exposure to hexavalent chromium. Environ Res. 2022;204(Pt A):111984.

90. Talaska G, Thoroman J, Schuman B, Käfferlein HU. Biomarkers of polycyclic aromatic hydrocarbon exposure in European coke oven workers. Toxicol Lett. 2014;231(2):213-6.

91. Tavares A, Aimonen K, Ndaw S, Fučić A, Catalán J, Duca RC, et al. HBM4EU Chromates Study-Genotoxicity and Oxidative Stress Biomarkers in Workers Exposed to Hexavalent Chromium. Toxics. 2022;10(8).

92. Vandebroek E, Haufroid V, Smolders E, Hons L, Nemery B. Occupational Exposure to Metals in Shooting Ranges: A Biomonitoring Study. Saf Health Work. 2019;10(1):87-94.

93. Wittsiepe J, Feldt T, Till H, Burchard G, Wilhelm M, Fobil JN. Pilot study on the internal exposure to heavy metals of informal-level electronic waste workers in Agbogbloshie, Accra, Ghana. Environ Sci Pollut Res Int. 2017;24(3):3097-107.

94. Ferland S, Cote J, Ratelle M, Thuot R, Bouchard M. Detailed Urinary Excretion Time Courses of Biomarkers of Exposure to Permethrin and Estimated Exposure in Workers of a Corn Production Farm in Quebec, Canada. Ann Occup Hyg. 2015;59(9):1152-67.

95. Hardt J, Angerer J. Biological monitoring of workers after the application of insecticidal pyrethroids. Int Arch Occup Environ Health. 2003;76(7):492-8.

96. Heinala M, Ylinen K, Tuomi T, Santonen T, Porras SP. Assessment of Occupational Exposure to Bisphenol A in Five Different Production Companies in Finland. Ann Work Expos Health. 2017;61(1):44-55.

97. Ndaw S, Leso V, Bousoumah R, Remy A, Bocca B, Duca RC, et al. HBM4EU chromates study- Usefulness of measurement of blood chromium levels in the assessment of occupational Cr(VI) exposure. Environ Res. 2022;214:10.

98. Schecter A, Colacino JA, Harris TR, Shah N, Brummitt SI. A Newly Recognized Occupational Hazard for US Electronic Recycling Facility Workers: Polybrominated Diphenyl Ethers. J Occup Environ Med. 2009;51(4):435-40.

99. Scheepers PTJ, Duca RC, Galea KS, Godderis L, Hardy E, Knudsen LE, et al. HBM4EU Occupational Biomonitoring Study on e-Waste-Study Protocol. Int J Environ Res Public Health. 2021;18(24):17.

100. Aribou ZM, Ng WT. Targeted medical examinations for workers exposed to fumigants. J Occup Med Toxicol. 2022;17(1):20.

101. Jaga K, Dharmani C. Ocular toxicity from pesticide exposure: A recent review. Environ Health Prev Med. 2006;11(3):102-7.

102. Pedroso TMA, Benvindo-Souza M, de Araújo Nascimento F, Woch J, Dos Reis FG, de Melo ESD. Cancer and occupational exposure to pesticides: a bibliometric study of the past 10 years. Environ Sci Pollut Res Int. 2022;29(12):17464-75.

103. Thompson RA, Sanderson WT, Westneat S, Bunn T, Lavender A, Tran A, et al. Perceptions of opioid and other illicit drug exposure reported among first responders in the southeast, 2017 to 2018. Health Sci Rep. 2021;4(3):e335.

104. World Health Organization. Depleted uranium : sources, exposure and health effects. Geneva: World Health Organization; 2001.

105. Georgia Department of Public Health. Georgia occupational health indicator: elevated blood lead levels (BLL) among adults, 2010-2015.

106. Jackson DA, Burr GA, Braun CR, de Perio MA. Notes from the Field: Lead Exposures Among Employees at a Bullet Manufacturing Company - Missouri, 2017. MMWR Morb Mortal Wkly Rep. 2018;67(39):1103.

107. Hughson GW. An occupational hygiene assessment of dermal inorganic lead exposures in primary and intermediate user industries. 2005.

108. Sewell C PABDTSNKMWCBRA. Epidemiological study of the relationships between exposure to organophosphate pesticides and indices of chronic peripheral neuropathy, and neuropsychological abnormalities in sheep farmers and dippers. Phase 1. Development and validation of an organophosphate uptake model for sheep dippers. 2006.

109. Rj Aitken KSCCLT. Nanoparticles: An occupational hygiene review. 2004.

110. Allaouat S, Reddy VK, Räsänen K, Khan S, Lumens M. Educational interventions for preventing lead poisoning in workers. Cochrane Database of Systematic Reviews. 2020(8).

111. Burton MJ, Clarkson JE, Goulao B, Glenny AM, McBain AJ, Schilder AGM, et al. Use of antimicrobial mouthwashes (gargling) and nasal sprays by healthcare workers to protect them when treating patients with suspected or confirmed COVID-19 infection. Cochrane Database of Systematic Reviews. 2020(9).

112. Amoah ID, Kumari S, Bux F. A probabilistic assessment of microbial infection risks due to occupational exposure to wastewater in a conventional activated sludge wastewater treatment plant. Sci Total Environ. 2022;843:156849.

113. Bakhmutsky MV, Oliver MS, McDiarmid MA, Squibb KS, Tucker JD. Long term depleted uranium exposure in Gulf War I veterans does not cause elevated numbers of micronuclei in peripheral blood lymphocytes. Mutat Res. 2011;720(1-2):53-7.

114. Chen MQ, Jiang JY, Gan ZW, Yan Y, Ding SL, Su SJ, et al. Grain size distribution and exposure evaluation of organophosphorus and brominated flame retardants in indoor and outdoor dust and PM10 from Chengdu, China. J Hazard Mater. 2019;365:280-8.

115. Damian P. Development of a health risk-based surface contamination cleanup standard for occupational exposure to beryllium. Toxicol Mech Methods. 2011;21(2):97-102.

116. Fang W, Yang Y, Xu Z. PM10 and PM2.5 and health risk assessment for heavy metals in a typical factory for cathode ray tube television recycling. Environ Sci Technol. 2013;47(21):12469-76.

117. Flack S, Nylander-French LA. Occupational chemicals: metabolism, toxicity, and mode of action. Prog Mol Biol Transl Sci. 2012;112:163-207.

118. Guney M, Zagury GJ. Bioaccessibility and other key parameters in assessing oral exposure to PAH-contaminated soils and dust: A critical review. Hum Ecol Risk Assess. 2016;22(6):1396-417.

119. Hu L, Bai L, Kang JN, Jia JL. Contamination level and potential health risk assessment of hexavalent chromium in soils from a coal chemical industrial area in Northwest China. Hum Ecol Risk Assess. 2020;26(5):1300-12.

120. Li Y, Chen L, Wen ZH, Duan YP, Lu ZB, Meng XZ, et al. Characterizing distribution, sources, and potential health risk of polybrominated diphenyl ethers (PBDEs) in office environment. Environ Pollut. 2015;198:25-31.

121. Perry MJ, Marbella A, Layde PM. Nonpersistent Pesticide Exposure Self-report versus Biomonitoring in Farm Pesticide Applicators. Annals of epidemiology. 2006;16(9):701-7.

122. Qin RX, Tang B, Zhuang X, Lei WX, Wang MH, Zhang LH, et al. Organophosphate flame retardants and diesters in the urine of e-waste dismantling workers: associations with indoor dust and implications for urinary biomonitoring. Environ Sci-Process Impacts. 2021;23(2):357-66.

123. Qu CS, Sun K, Wang SR, Huang L, Bi J. Monte Carlo Simulation-Based Health Risk Assessment of Heavy Metal Soil Pollution: A Case Study in the Qixia Mining Area, China. Hum Ecol Risk Assess. 2012;18(4):733-50.

124. Ritchie GD, Still KR, Alexander WK, Nordholm AF, Wilson CL, Rossi J, et al. A review of the neurotoxicity risk of selected hydrocarbon fuels. J Toxicol Env Health-Pt b-Crit Rev. 2001;4(3):223-312.

125. Roldán-Tapia L, Parrón T, Sánchez-Santed F. Neuropsychological effects of long-term exposure to organophosphate pesticides. Neurotoxicol Teratol. 2005;27(2):259-66.

126. Waheed S, Halsall C, Sweetman AJ, Jones KC, Malik RN. Pesticides contaminated dust exposure, risk diagnosis and exposure markers in occupational and residential settings of Lahore, Pakistan. Environ Toxicol Pharmacol. 2017;56:375-82.

127. Barker SF, O'Toole J, Sinclair MI, Keywood M, Leder K. Endotoxin health risk associated with high pressure cleaning using reclaimed water. Microb Risk Anal. 2017;5:65-70.

128. Chiang KC, Chio CP, Chiang YH, Liao CM. Assessing hazardous risks of human exposure to temple airborne polycyclic aromatic hydrocarbons. J Hazard Mater. 2009;166(2-3):676-85.

129. Gulson BL, Mizon KJ, Dickson BL, Korsch MJ. The effect of exposure to employees from mining and milling operations in a uranium mine on lead isotopes--a pilot study. Sci Total Environ. 2005;339(1-3):267-72.

130. Jones-Otazo HA, Clarke JP, Diamond ML, Archbold JA, Ferguson G, Harner T, et al. Is house dust the missing exposure pathway for PBDEs? An analysis of the urban fate and human exposure to PBDEs. Environ Sci Technol. 2005;39(14):5121-30.

131. Kamal A, Malik RN, Martellini T, Cincinelli A. Cancer risk evaluation of brick kiln workers exposed to dust bound PAHs in Punjab province (Pakistan). Sci Total Environ. 2014;493:562-70.

132. Krzyzanowski F, Jr., de Souza Lauretto M, Nardocci AC, Sato MIZ, Razzolini MTP. Assessing the probability of infection by Salmonella due to sewage sludge use in agriculture under several exposure scenarios for crops and soil ingestion. Sci Total Environ. 2016;568:66-74.

133. Li J, Gao X, He Y, Wang L, Wang Y, Zeng L. Elevated emissions of melamine and its derivatives in the indoor environments of typical e-waste recycling facilities and adjacent communities and implications for human exposure. J Hazard Mater. 2022;432:128652.

134. Liu NN, Xu L, Cai YQ. Methyl siloxanes in barbershops and residence indoor dust and the implication for human exposures. Sci Total Environ. 2018;618:1324-30.

135. Liu X, Cao Z, Yu G, Wu M, Li X, Zhang Y, et al. Estimation of Exposure to Organic Flame Retardants via Hand Wipe, Surface Wipe, and Dust: Comparability of Different Assessment Strategies. Environ Sci Technol. 2018;52(17):9946-53.

136. Lurker PA, Berman F, Clapp RW, Stellman JM. Post-Vietnam military herbicide exposures in UC-123 Agent Orange spray aircraft. Environ Res. 2014;130:34-42.

137. Othman M, Latif MT, Mohamed AF. Health impact assessment from building life cycles and trace metals in coarse particulate matter in urban office environments. Ecotoxicol Environ Saf. 2018;148:293-302.

138. Ali N, Ismail IMI, Khoder M, Shamy M, Alghamdi M, Al Khalaf A, et al. Polycyclic aromatic hydrocarbons (PAHs) in the settled dust of automobile workshops, health and carcinogenic risk evaluation. Sci Total Environ. 2017;601:478-84.

139. Fernández-Caliani JC, Giráldez MI, Barba-Brioso C. Oral bioaccessibility and human health risk assessment of trace elements in agricultural soils impacted by acid mine drainage. Chemosphere. 2019;237:124441.

140. Zou YP, Li YH, Hu L, Yang S, Zhang K, Kang JN, et al. Health risk assessment of arsenic in soils from three thermal power plants in Southwest China. Hum Ecol Risk Assess. 2020;26(5):1221-33.

141. Xia M, Ouyang X, Wang X, Shen X, Zhan Y. Occupational exposure assessment of phthalate esters in indoor and outdoor microenvironments. J Environ Sci (China). 2018;72:75-88.

142. Zhang M, Shi J, Meng Y, Guo W, Li H, Liu X, et al. Occupational exposure characteristics and health risk of PBDEs at different domestic e-waste recycling workshops in China. Ecotoxicol Environ Saf. 2019;174:532-9.

143. Zou YP, Liu JL, Liu XC, Jia JL. Health risk assessment of polycyclic aromatic hydrocarbons (PAHs) in the soil around thermal power plants in southwest China. J Environ Sci Health Part A-Toxic/Hazard Subst Environ Eng. 2021;56(7):786-96.

144. Guo J, Zhou Y, Cui J, Zhang B, Zhang J. Assessment of volatile methylsiloxanes in environmental matrices and human plasma. Sci Total Environ. 2019;668:1175-82.

145. Shen M, Ge J, Lam JCW, Zhu M, Li J, Zeng L. Occurrence of two novel triazine-based flame retardants in an E-waste recycling area in South China: Implication for human exposure. Sci Total Environ. 2019;683:249-57.

146. Wannomai T, Matsukami H, Uchida N, Takahashi F, Tuyen LH, Viet PH, et al. Bioaccessibility and exposure assessment of flame retardants via dust ingestion for workers in e-waste processing workshops in northern Vietnam. Chemosphere. 2020;251:126632.

147. Shen YW, Zhao H, Xie JJ, He KQ, Pang JF, Guo Q, et al. Insight of the size dependent bioavailability and health risk assessment of arsenic in resuspended fly ash from power plants. Fuel. 2022;327:9.

148. Gao P, Liu S, Ye WY, Lin N, Meng P, Feng YJ, et al. Assessment on the occupational exposure of urban public bus drivers to bioaccessible trace metals through resuspended fraction of settled bus dust. Sci Total Environ. 2015;508:37-45.

149. Gerritsen-Ebben MG, Brouwer DH, van Hemmen JJ. Personal protective equipment for registration purposes of pesticides. Commun Agric Appl Biol Sci. 2007;72(2):87-93.

150. Die Q, Nie Z, Huang Q, Yang Y, Fang Y, Yang J, et al. Concentrations and occupational exposure assessment of polybrominated diphenyl ethers in modern Chinese e-waste dismantling workshops. Chemosphere. 2019;214:379-88.

151. Zhou X, Guo J, Zhang W, Zhou P, Deng J, Lin K. Tetrabromobisphenol A contamination and emission in printed circuit board production and implications for human exposure. J Hazard Mater. 2014;273:27-35.
